# An Unusual Case of Anderson-Fabry Disease: Case Report

**DOI:** 10.2196/49573

**Published:** 2024-01-16

**Authors:** Alpana Mohta, Achala Mohta, Pramila Kumari

**Affiliations:** 1 Department of Dermatology, Venereology and Leprosy Sardar Patel Medical College Bikaner India; 2 Department of Preventive and Social Medicine Sardar Patel Medical College Bikaner India; 3 Department of Dermatology Era's Lucknow Medical College Lucknow India

**Keywords:** angiokeratoma, Fabry disease, angiokeratoma corporis diffusum, vascular, capillary, capillaries, blood vessel, lysosome, lysosomal, enzyme, enzymatic, case report, circulatory, skin, dermatology, dermatological

## Abstract

Angiokeratoma is a group of capillary malformations characterized by the formation of variably sized dark red hyperkeratotic papules. Initially, it was believed that angiokeratoma corporis diffusum was a telltale sign of Anderson-Fabry disease; however, current consensus states that it is also seen in various other lysosomal enzymatic deficiencies. In this report, we present the case of a 12-year-old boy who developed angiokeratoma corporis diffusum with sensorineural deafness, acroparesthesias, and renal involvement.

## Introduction

Angiokeratoma is a group of capillary malformations characterized by the formation of variably sized dark red hyperkeratotic papules. The capillaries are dilated in the papillary dermis with reactionary epidermal hyperplasia and hyperkeratosis. Clinically, various patterns of angiokeratoma have been identified, namely, solitary isolated or multiple angiokeratomas, angiokeratoma of Fordyce, angiokeratoma corporis diffusum, angiokeratoma circumscripta, and angiokeratoma of Mibelli. The generalized variant of angiokeratoma is known as angiokeratoma corporis diffusum [1,2].

Initially, it was believed that angiokeratoma corporis diffusum is a telltale sign of Anderson-Fabry disease, but current consensus states that it is also seen in various other lysosomal enzymatic deficiencies. In this case report, we present the case of a 12-year-old boy who developed angiokeratoma corporis diffusum with sensorineural deafness, acroparesthesias, and renal involvement.

## Case Report

**Figure 1:**
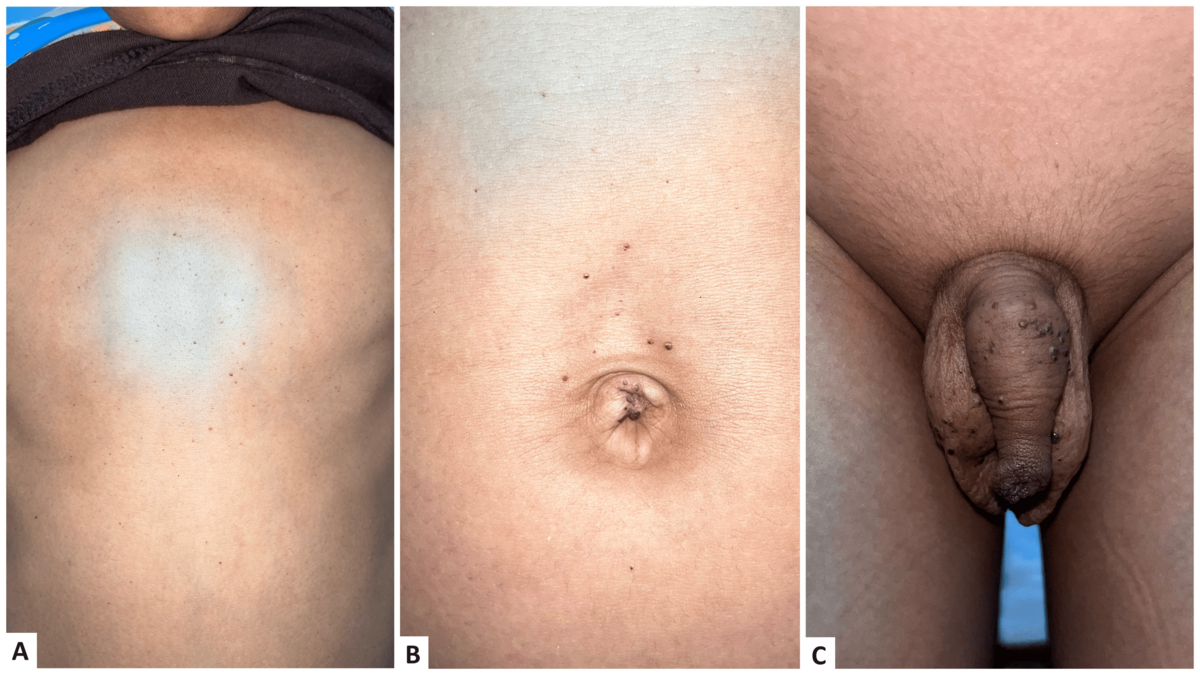
(A) discreet angiokeratoma over trunk; (B) clustered angiokeratoma over umbilicus; (C) clustered angiokeratoma over genitalia.

A 12-year-old boy with average intelligence presented to us with multiple pinhead-sized dark red papular eruptions all over his body since the age of 6. The lesions first appeared on the legs and gradually increased over several years, involving bilateral limbs and trunks, with clustering over the genitalia (Figure 1A, 1B, and 1C). Upon examination, discreet and grouped nonblanchable angiomatous papules were observed, distributed symmetrically across the entire body, with relative sparing of the face, palms, soles, and mucosa. There was the presence of hyperkeratosis over some of the angiomatous papules.

The patient confirmed that the lesions would bleed when scratched. The patient also reported experiencing generalized asthenia and a low-grade fever 4 months prior. On further inquiry, the patient revealed that he had shooting pains starting from the back and radiating to bilateral lower limbs for the past 3 months. However, until his current visit, his family had not sought any treatment for his condition.

The child had no history of seizures, visual disturbances, hearing loss, or atypical facial features. There was no history of similar skin lesions or associated features in any family members. The child also had bilateral cervical lymphadenopathy. On pure tone audiometry, there was sensorineural hearing loss in both ears. No ocular abnormalities were detected on the slit lamp and fundus examination. Lab investigations revealed microcytic hypochromic anemia, thrombocytopenia, and hypoproteinemia. On further biochemical analysis, the child’s leukocyte α-Galactosidase A activity was very low (0.1 nmol/h/mL). The child’s galactosidase alpha gene study revealed a missense mutation in α-Galactosidase A. The remaining investigations and imaging (ie, electrocardiogram, high-resolution computed tomography, ultrasonography, and chest x-ray) were unremarkable.

Due to the patient’s low socioeconomic status, a genetic study could not be carried out for the rest of the family members.

**Figure 2:**
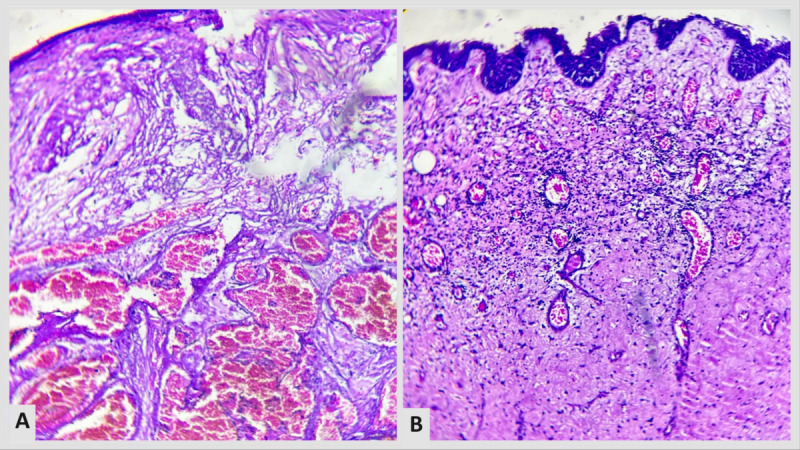
(A) hyperkeratosis with ectatic dermal blood vessels (hematoxylin and eosin; 100 times magnification under a microscope); (B) dilated capillaries lined by vacuolated endothelium.

Upon histopathological evaluation, thin-walled ectatic capillaries having vacuolated endothelial cells were observed in the upper dermis. The epidermis had elongated rete ridges and hyperkeratosis (Figure 2A and 2B). Enzyme assay could not be done due to resource limitations and financial constraints. A diagnosis of angiokeratoma corporis diffusum was made. The course and prognosis of the disease were explained to the patient and his family. The large angiokeratomas were removed using radiofrequency ablation, and the patient is currently being managed with a multidisciplinary approach, including intravenous α-Galactosidase A enzyme replacement therapy infusion. The case is still being followed up with a measure of improvement in his acroparesthesia following 3 months of treatment.

## Discussion

Angiokeratoma corporis diffusum was described for the first time in 1898. Although Angiokeratoma corporis diffusum has often been used interchangeably with Anderson-Fabry disease, the latter may be associated with lysosomal defects, including fucosidosis, mannosidosis, sialidosis, Kanzaki disease, and monosialotetrahexosylganglioside gangliosidosis [3,4]. Anderson-Fabry disease is an X-linked disorder. In this disease, there is a deficiency in the enzyme α-Galactosidase A, which is responsible for glycosphingolipid catabolism. This deficiency leads to the accumulation of glycosphingolipids, chiefly globotriaosylceramide (GL3) and a metabolite of GL3 called globotriaosylsphingosine (lyso-GL3) in various cells. This accumulation predominantly affects the kidney, heart, and nervous system, contributing to systemic involvement [5].

Fabry disease mutations are observed in around 1 in 22,000-40,000 male individuals, whereas atypical presentations are linked to approximately 1 in 1000-3000 male and 1 in 6000-40000 female individuals [6].

This condition can be categorized into 2 main types: a severe classical form, typically observed in men with no residual enzyme activity, and a milder nonclassical form. Classical Fabry disease is associated with neuropathic pain, cornea verticillate, and angiokeratoma. Over time, it can lead to issues like cardiac rhythm problems, hypertrophic cardiomyopathy, progressive renal failure, and stroke.

On the other hand, nonclassical Fabry disease, also known as late-onset or atypical Fabry disease, displays a more variable progression. Patients with this form are generally less severely affected, and their symptoms may be confined to 1 organ. Despite its X-linked inheritance pattern, women can also experience Fabry disease symptoms, but their condition is typically less severe than that of men due to X-inactivation patterns in women [7].

Often, acroparesthesia in Anderson-Fabry disease is precipitated by emotional or physical stress, febrile illness, and prolonged temperature variation [8]. In our patient, acroparaesthesia was triggered by an episode of febrile illness.

Our patient also had hypoalbuminemia, an indicator of renal disease. Kidneys are one of the most commonly involved organs in Anderson-Fabry disease, often resulting in end-stage renal disease and a high mortality rate in untreated patients. Manifestations often mirror diabetic nephropathy’s progression—initial hyperfiltration, followed by albuminuria, heavy proteinuria, and gradual kidney function decline. Tubular manifestations, though rarer, involve renal tubular acidosis, Fanconi syndrome, and impaired urine concentration. Renal involvement is attributed to GL3-induced inflammation and oxidative damage to the glomeruli and podocytes in the kidneys [9].

Fabry disease has no complete cure. To manage it, enzyme replacement (α-Galactosidase A) is initiated upon diagnosis, irrespective of symptoms in affected male patients or those on renal therapy. Female carriers and male patients with low α-Galactosidase A levels receive enzyme replacement only if they exhibit kidney, neurological, or heart issues. Patients with a history of long-term dialysis also receive enzyme replacement. Hypertension is managed with medications like angiotensin-converting enzyme inhibitors or angiotensin receptor blockers. Enzyme infusions (alpha or beta) are administered every 2 weeks based on body weight [6].

## Conclusions

This report highlights the high reliability of a thorough clinical evaluation for diagnosing atypical and unusual variants of genodermatoses, including Anderson-Fabry disease. Angiokeratoma is a reliable clinical indicator when screening patients for Anderson-Fabry disease. Early identification of these lesions aids in early detection of the disease, enabling timely treatment.

## Abbreviations

GL3: globotriaosylceramide

## Data Availability

The data that support the findings of this series are available from the corresponding author upon reasonable request.
